# Supplementary material for: Neurokinin-3 receptor activation selectively prolongs atrial refractoriness by inhibition of a background K+ channel
Source: Nat Commun. 2018 Oct 19;9:4357. doi: 10.1038/s41467-018-06530-5 (PMC6195571; doi:10.1038/s41467-018-06530-5)
Supplement: Supplementary file 1 — Supplementary Information [file 41467_2018_6530_MOESM1_ESM.pdf]

Neurokinin-3 receptor activation prolongs atrial refractoriness by inhibition of a background K^+ channel

Veldkamp et al.

Supplementary FIGURES

Supplementary Figure 1

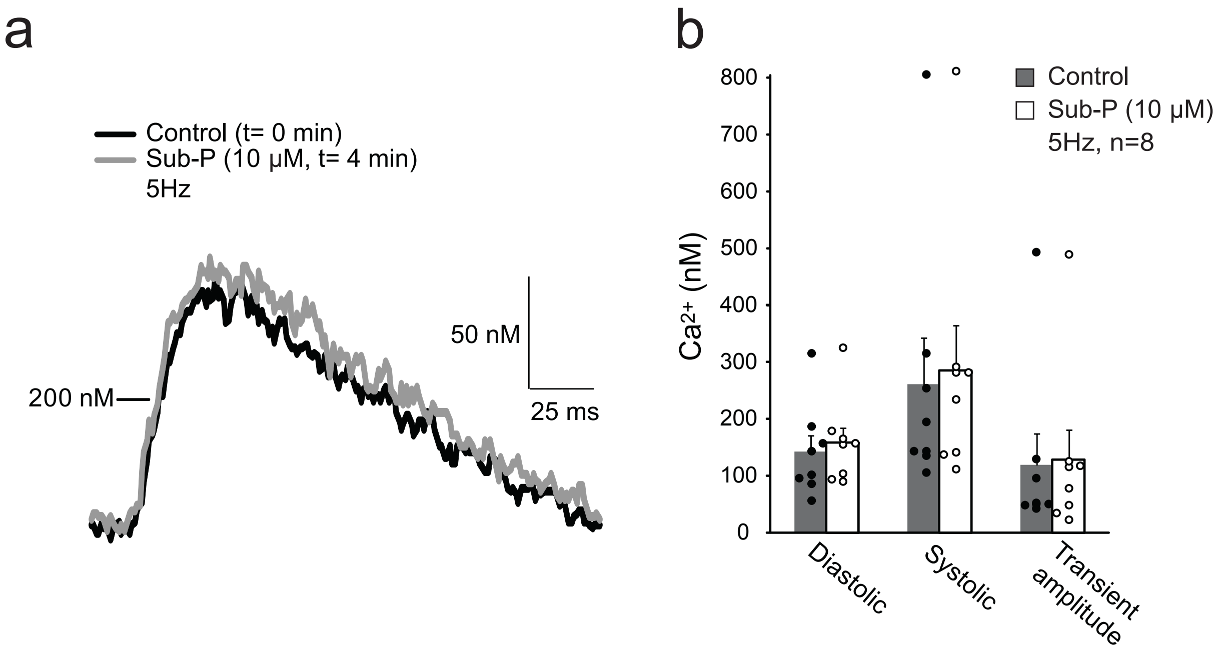

SUPPLEMENTARY FIGURE 1. Effect of Substance-P on intracellular calcium concentration. (**a**) Intracellular calcium (Ca^{2+}_i) transients in Control conditions and in the presence of 10 µmol/L Sub-P. (**b**) Bar histograms showing average diastolic, systolic and peak Ca^{2+}_i before (Control) and after application of 10 µM Sub-P (n=8). All values shown are mean±SEM. Paired *t*-test. * P<0.05

Supplementary Figure 2

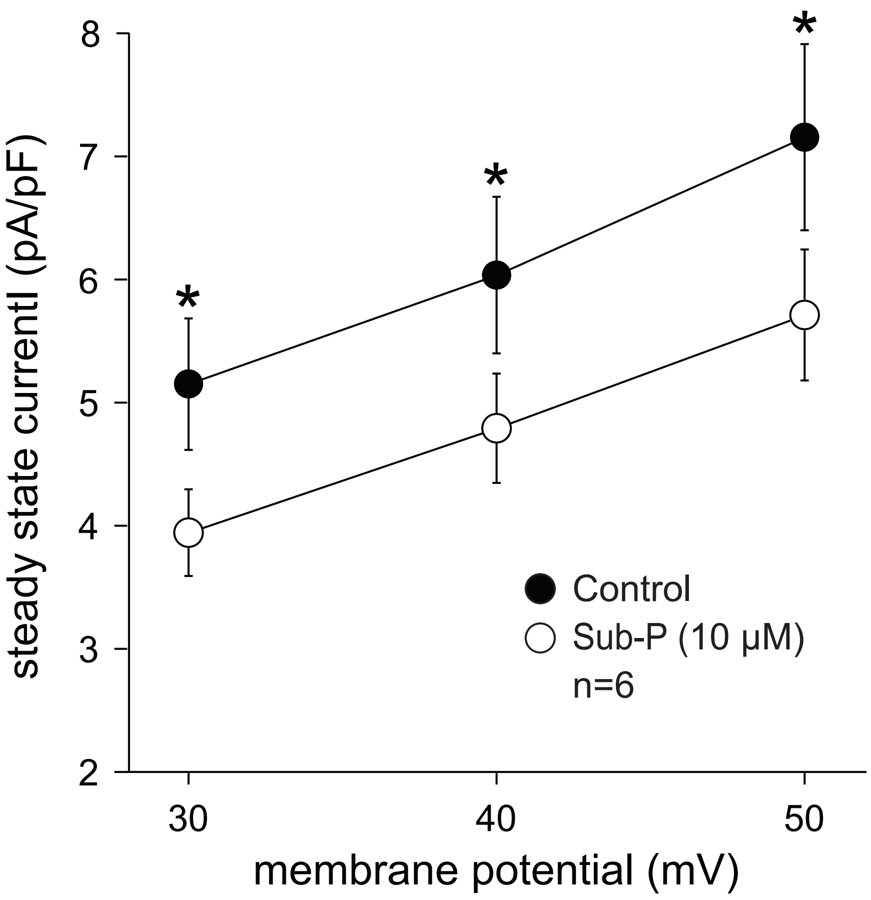

SUPPLEMENTARY FIGURE 2. Effect of neurokinin-3 receptor (NK-3R) stimulation on steady-state currents in the presence of blockers for Na^+-, transient outward and ultra-rapid delayed rectifier K^+ currents. Average current-voltage relationships of steady-state current at t=300 ms before (Control) and after application of 10 μM Sub-P (n=6). All values shown are mean±SEM. Two-way RM ANOVA. * P<0.05.

Supplementary Figure 3.

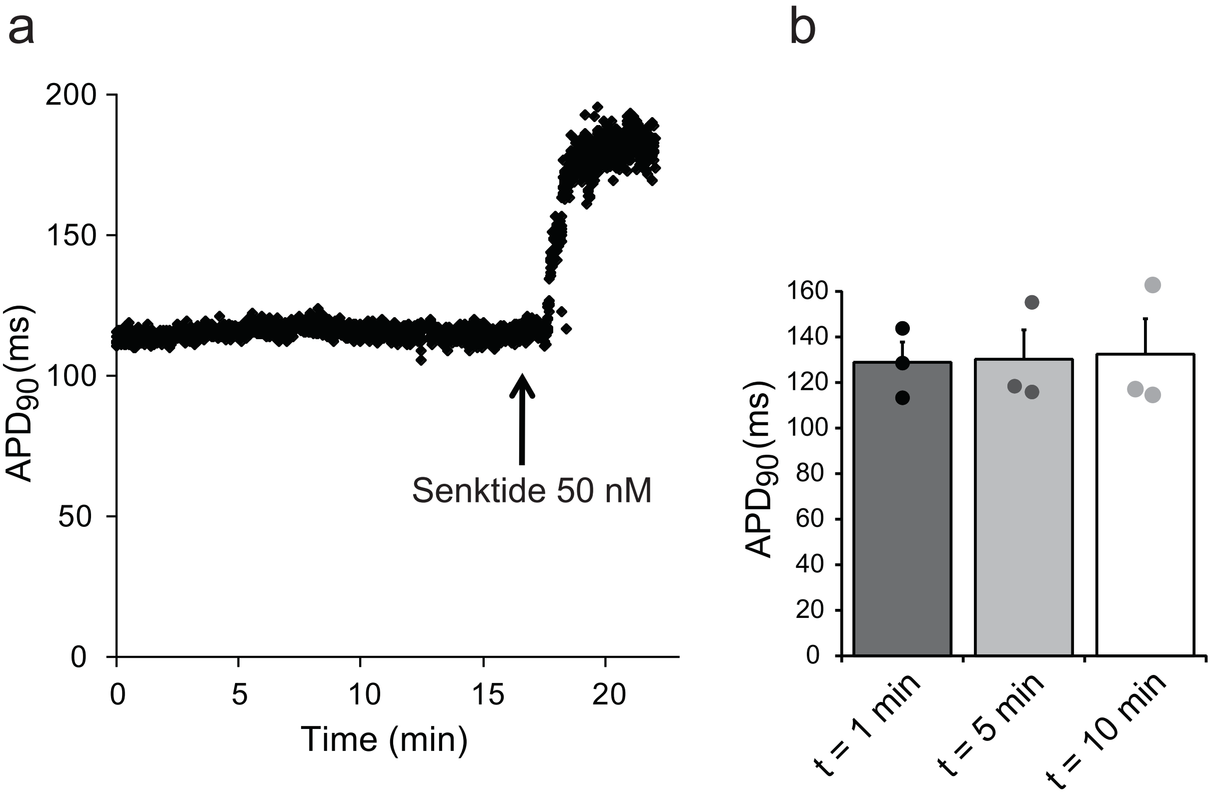

SUPPLEMENTARY FIGURE 3. Action potential duration is stable in time. (**a**) Action potential duration at 90% of repolarization (APD_{90}) as a function of time. At t=16 min. 50 nM Senktide was applied. (**b**) APD_{90} at 1, 5, and 10 min after the start of the experiment (n=3). All values shown are mean±SEM. Paired *t*-test. * P<0.05.

.

Supplementary Figure 4

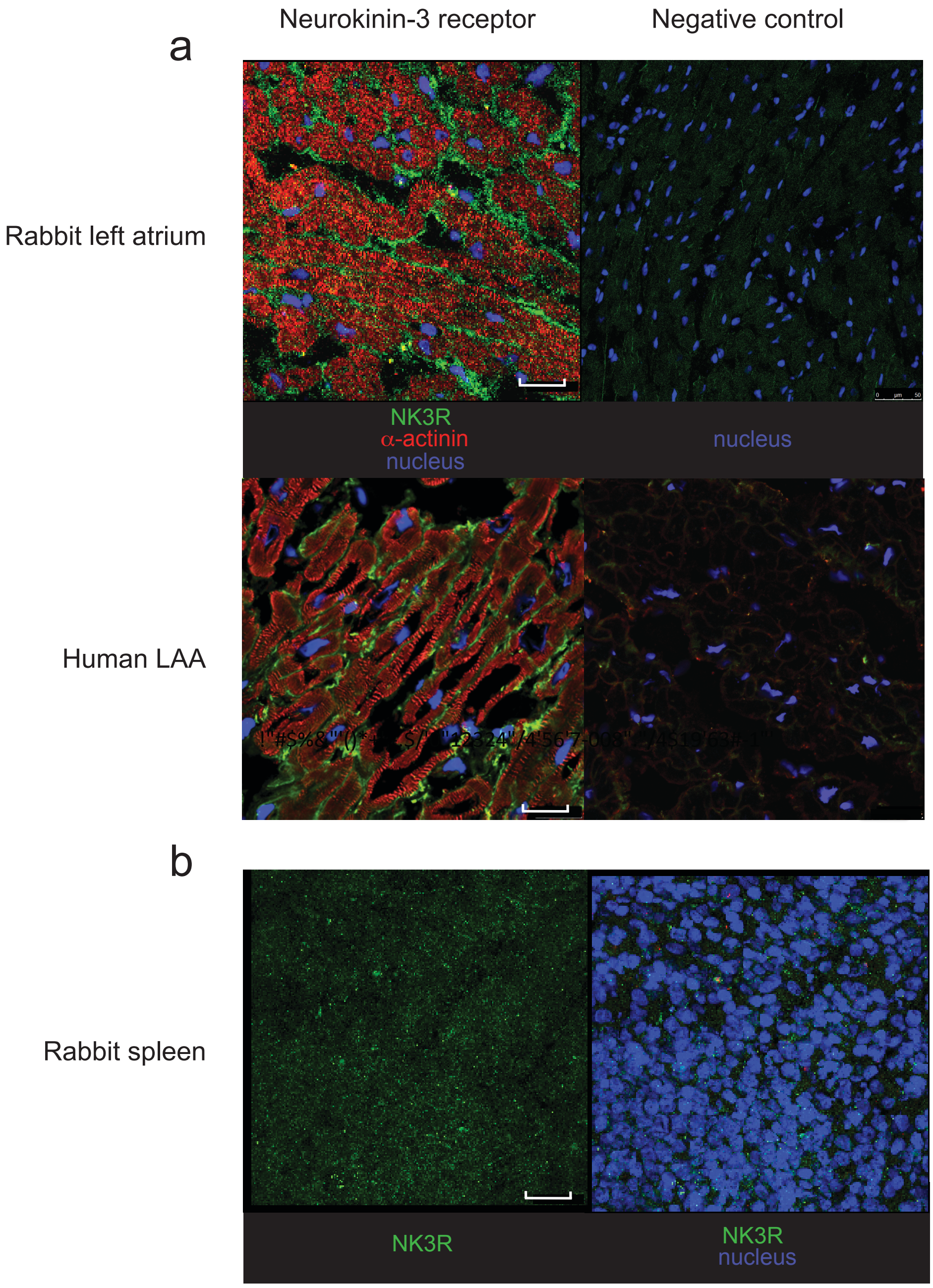

SUPPLEMENTARY FIGURE 4. Negative controls for NK-3R staining in rabbit and human atrium. (**a**) NK-3R positive staining (green) in tissue sections of rabbit left atrium (**top, left**) and human LAA (**bottom, left**). In rabbit sections, the fluorophore was conjugated to the primary antibody directly. In human sections, the fluorophore was conjugated to the secondary antibody. (**Top, right**) No primary antibody controls for rabbit and human (**bottom, right**). Tissue sections were incubated with secondary antibody only. (**b**) A section from rabbit spleen incubated with fluorophore-conjugated primary NK-3R antibody. Spleen has been reported not to express the target antigen NK-3R[1].

Supplementary Figure 5

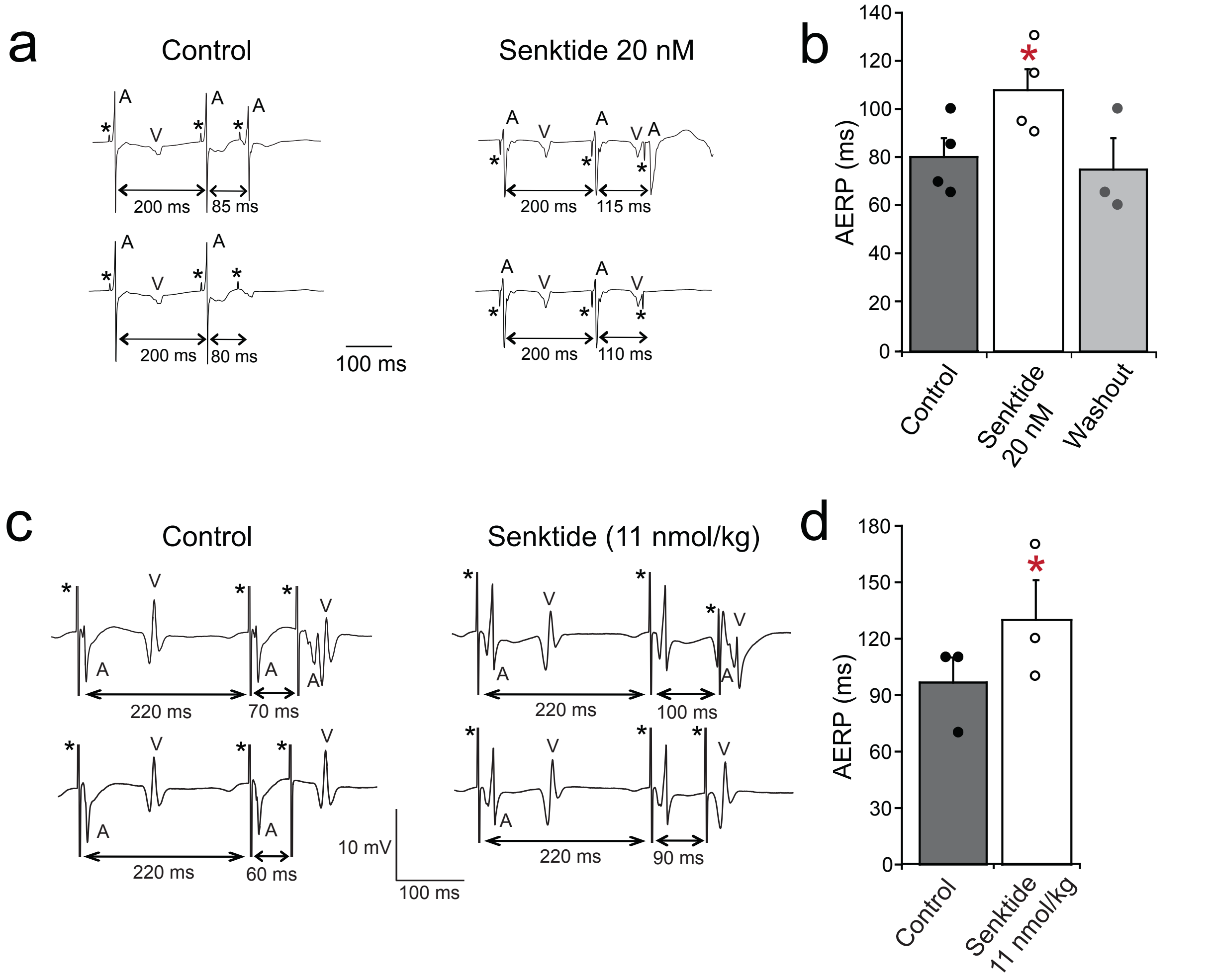

SUPPLEMENTARY FIGURE 5. . Effect of neurokinin-3 receptor (NK-3R) stimulation on effective refractory period (AERP) in rabbit intact left atrium *ex vivo* and *in situ*. (**a**) Unipolar electrograms recorded from the left atrium of a rabbit Langendorff perfused heart before (Control) and after application of 20 nM of the NK-3R agonist Senktide. (**b**) Bar graph showing average values for left AERP in before (Control) and after application of 20 nM Senktide (n=4). (**c**) Unipolar electrograms recorded from the left atrium of the *in situ* rabbit heart in before (Control) and after infusion of 11 nmol/kg of Senktide. (**d**) Bar graph showing average values for AERP before (Control) and after infusion of 11 nmol/kg Senktide (n=3). All values shown are mean±SEM. Two-way RM ANOVA. * P<0.05

Electograms: A, atrial activation; V, ventricular activation; *, stimulus.

Supplementary Table 1. Effects of selective TASK-1, TASK-3, TREK-1 channel blockers, and Senktide on action potential parameters in atrial myocytes

	**RMP (mV)**	**APA (mV)**	**Vmax (V/s)**	**APD_{20} (ms)**	**APD_{50} (ms)**	**APD_{90} (ms)**
**Atrial myocyte**						
Control	-83.0±0.9	110.5±2.1	357.2 ±29.1	4.9 ±0.6	23.3±5.0	95.6 ±10.5
ML-365 (100 nM, n=8)	-83.5±1.0	99.2±12.7	382.8±30.8	5.8±1.0	27.2±5.3*	100.4±10.9*
ML-365 + Senktide (100 nM, 50 nM, n=8)	-83.6±0.7	110.3±2.5	357.9±31.4	7.9±2.0	48.9±6.9*#	139.0±15.9*#
Control	-81.2±0.9	104.2±1.9	341.6 ±30.9	4.0 ±0.6	14.0 ±4.7	73.5 ±3.2
PK-THPP (100 nM, n=7)	-80.5±0.4	103.1±2.1	322.2±28.2	4.5±0.7*	17.9±5.4*	79.0±3.1*
PK-THPP + Senktide (100 nM, 50 nM, n=7)	-78.2±2.3	95.8±4.7	250.6±47.8	5.2±0.4*	40.8±8.6*#	119.1±5.1*#
Control	-82.4±1.5	107.2±3.7	168.6 ±36.7	7.2 ±1.0	26.1±4.6	80.1 ±6.7
Spadin (100 nM, n=7)	-82.8±1.6	108.3±4.1	193.7±41.5	6.2±1.0	27.3±4.7	82.1±6.8
Spadin + Senktide (100 nM, 50 nM, n=7)	-81.2±2.6	105.4±6.0	162.4±46.0	8.1±1.6	48.2±8.4*#	114.6±13.0*#

All values are mean±SEM. RMP, resting membrane potential; APA, action potential amplitude; Vmax, upstroke velocity; APD20, action potential duration at 20% repolarization; APD50, action potential duration at 50% repolarization; APD90, action potential duration at 90% repolarization. One-way RM ANOVA followed by post hoc test. *p<0.05 vs. Control; #p<0.05 vs. Blocker ML-365, PK-THPP or Spadin, respectively.

Supplementary Table 2. Effects of Senktide on incidence and duration of atrial fibrillation (AF) in a rabbit isolated heart model of AF based on atrial dilatation

	Control		Senktide (20 nM)		Wash out	
**Incidence**	0 cm H_2O	20 cm H_2O	0 cm H_2O	20 cm H_2O	0 cm H_2O	20 cm H_2O
No Arrhythmia	0.23±0.06	0.11±0.04	0.46±0.06$^{\#}$	0.39±0.14$^{\$}$	0.24±0.08	0.02±0.02
Arrhythmia 0 < Δt > 1 s	0.69±0.05	0.30±0.07	0.54±0.06	0.44±0.10	0.66±0.07	0.34±0.05
AF Episode Δt = >1 s	0.08±0.04	0.59±0.09*	0.0±0.0$^{\#}$	0.17±0.08*$^{\$}$	0.10±0.08	0.64±0.07*
**Duration**						
AF Total duration	4.0±0.6	20.6±5.3*	1.5±0.2	6.6±2.5*$^{\$}$	3.7±1.0	30.0±8.7*

All values are mean ± SEM. Two-way RM ANOVA. *$p<0.05$ vs. 0 cm H_2O within each group (Control, Senktide, Wash out). \$$p<0.05$ vs. 20 cm H_2O in Control and Wash out. #$p<0.05$ vs. 0 cm H_2O in Control and Wash out.

Supplementary References:

1. Pinto F.M., Almeida T.A., Hernandez M., Devillier P, Advenier C., Candenas M.L. mRNA expression of tachykinins and tachykinin receptors in different human tissues. *Eur J Pharmacol*. 494, 233–239 (2004). doi:10.1016/j.ejphar.2004.05.016
